# What Do We Have to Know about PD-L1 Expression in Prostate Cancer? A Systematic Literature Review. Part 4: Experimental Treatments in Pre-Clinical Studies (Cell Lines and Mouse Models)

**DOI:** 10.3390/ijms222212297

**Published:** 2021-11-14

**Authors:** Andrea Palicelli, Stefania Croci, Alessandra Bisagni, Eleonora Zanetti, Dario De Biase, Beatrice Melli, Francesca Sanguedolce, Moira Ragazzi, Magda Zanelli, Alcides Chaux, Sofia Cañete-Portillo, Maria Paola Bonasoni, Alessandra Soriano, Stefano Ascani, Maurizio Zizzo, Carolina Castro Ruiz, Antonio De Leo, Guido Giordano, Matteo Landriscina, Giuseppe Carrieri, Luigi Cormio, Daniel M. Berney, Jatin Gandhi, Giacomo Santandrea, Martina Bonacini

**Affiliations:** 1 Pathology Unit, Azienda USL-IRCCS di Reggio Emilia, 42123 Reggio Emilia, Italy; Alessandra.Bisagni@ausl.re.it (A.B.); Eleonora.Zanetti@ausl.re.it (E.Z.); Moira.Ragazzi@ausl.re.it (M.R.); Magda.Zanelli@ausl.re.it (M.Z.); mariapaola.bonasoni@ausl.re.it (M.P.B.); giacomo.santandrea@ausl.re.it (G.S.); 2 Clinical Immunology, Allergy and Advanced Biotechnologies Unit, Azienda USL-IRCCS di Reggio Emilia, 42123 Reggio Emilia, Italy; Stefania.Croci@ausl.re.it (S.C.); Martina.Bonacini@ausl.re.it (M.B.); 3 Department of Pharmacy and Biotechnology (FABIT), University of Bologna, 40126 Bologna, Italy; dario.debiase@unibo.it; 4 Fertility Center, Department of Obstetrics and Gynecology, Azienda USL-IRCCS di Reggio Emilia, 42123 Reggio Emilia, Italy; Beatrice.Melli@ausl.re.it; 5 International Doctorate School in Clinical and Experimental Medicine, University of Modena and Reggio Emilia, 41121 Modena, Italy; Carolina.CastroRuiz@ausl.re.it; 6 Pathology Unit, Policlinico Riuniti, University of Foggia, 71122 Foggia, Italy; francesca.sanguedolce@unifg.it; 7 Department of Scientific Research, School of Postgraduate Studies, Norte University, Asunción 1614, Paraguay; alcideschaux@uninorte.edu.py; 8 Department of Pathology, University of Alabama at Birmingham, Birmingham, AL 35294, USA; scaneteportillo@uabmc.edu; 9 Department of Pathology, Case Western Reserve University, Cleveland, OH 44106, USA; alessandra.soriano@ausl.re.it; 10 Gastroenterology Division, Azienda USL-IRCCS di Reggio Emilia, 42123 Reggio Emilia, Italy; 11 Pathology Unit, Azienda Ospedaliera Santa Maria di Terni, University of Perugia, 05100 Terni, Italy; s.ascani@aospterni.it; 12 Haematopathology Unit, CREO, Azienda Ospedaliera di Perugia, University of Perugia, 06129 Perugia, Italy; 13 Surgical Oncology Unit, Azienda USL-IRCCS di Reggio Emilia, 42123 Reggio Emilia, Italy; Maurizio.Zizzo@ausl.re.it; 14 Molecular Diagnostic Unit, Azienda USL Bologna, Department of Experimental, Diagnostic and Specialty Medicine, University of Bologna, 40138 Bologna, Italy; antonio.deleo@unibo.it; 15 Medical Oncology Unit, Department of Medical and Surgical Sciences, University of Foggia, 71122 Foggia, Italy; guido.giordano@unifg.it (G.G.); matteo.landriscina@unifg.it (M.L.); 16 Department of Urology and Renal Transplantation, University of Foggia, 71122 Foggia, Italy; giuseppe.carrieri@unifg.it (G.C.); luigi.cormio@unifg.it (L.C.); 17 Barts Cancer Institute, Queen Mary University of London, London EC1M 5PZ, UK; daniel.berney@nhs.net; 18 Department of Pathology and Laboratory Medicine, University of Washington, Seattle, WA 98195, USA; jgandhi@uw.edu

**Keywords:** PD-L1, prostate, cancer, signaling pathways, microenvironment, target-therapy, immunotherapy, checkpoint inhibitors

## Abstract

In prostate cancer (PC), the PD-1/PD-L1 axis regulates various signaling pathways and it is influenced by extracellular factors. Pre-clinical experimental studies investigating the effects of various treatments (alone or combined) may discover how to overcome the immunotherapy-resistance in PC-patients. We performed a systematic literature review (PRISMA guidelines) to delineate the landscape of pre-clinical studies (including cell lines and mouse models) that tested treatments with effects on PD-L1 signaling in PC. NF-kB, MEK, JAK, or STAT inhibitors on human/mouse, primary/metastatic PC-cell lines variably down-modulated PD-L1-expression, reducing chemoresistance and tumor cell migration. If PC-cells were co-cultured with NK, CD8+ T-cells or CAR-T cells, the immune cell cytotoxicity increased when PD-L1 was downregulated (opposite effects for PD-L1 upregulation). In mouse models, radiotherapy, CDK4/6-inhibitors, and *RB* deletion induced PD-L1-upregulation, causing PC-immune-evasion. Epigenetic drugs may reduce PD-L1 expression. In some PC experimental models, blocking only the PD-1/PD-L1 pathway had limited efficacy in reducing the tumor growth. Anti-tumor effects could be increased by combining the PD-1/PD-L1 blockade with other approaches (inhibitors of tyrosine kinase, PI3K/mTOR or JAK/STAT3 pathways, p300/CBP; anti-RANKL and/or anti-CTLA-4 antibodies; cytokines; nitroxoline; DNA/cell vaccines; radiotherapy/Radium-223).

## 1. Introduction

The multi-step process of cancerogenesis and tumor progression implies the acquirement of sustained proliferative signaling, evading growth suppression, reprogramming energy metabolism, and enabling replicative immortality, as well as the induction of angiogenesis and the promotion of invasion and metastatic dissemination; moreover, the escape of tumor cells from immune destruction plays an important role in cancer progression [1].

The establishment of an immunosuppressive microenvironment favoring tumor growth is a fundamental strategy for tumor immune evasion through the activation and production of suppressive immune cells (regulatory T-cells, myeloid cells, dendritic cells, etc.), the inhibition of antigen-presenting machinery, the production of immunosuppressive mediators, and the induction of anergy or apoptosis of cytotoxic immune cells [1]. 

As the discovery of novel biomarkers is urgently required to develop tailored therapies for various malignancies [2], increasing attention has been paid to immunotherapy targets such as Programmed death-1 (PD-1) and its ligand (PD-L1). This pathway is involved in tumor immune-escape and it can be targeted by drugs recently approved by the Food and Drug Administration. Indeed, Pembrolizumab monotherapy (anti-PD-1 monoclonal antibody) recently revealed good therapeutic activity, and the 2021 United States National Comprehensive Cancer Network (NCCN) guidelines have considered this drug indicated in selected prostate cancer (PC) patients [3,4]. PD-1 and PD-L1 are type I transmembrane glycoproteins transcribed by *PDCD1* (located on chromosome 2) and *CD274* genes (located on chromosome 9), respectively [5,6]. PD-1 is expressed by activated T, B, NK cells and monocytes, while PD-L1 is found on hematopoietic and non-hematopoietic cells [5,6]. 

The PD-1/PD-L1 axis activates multiple molecular pathways, and it is influenced by other various signaling pathways, as well as by extracellular factors: this complex regulation makes the predictivity of response to treatment difficult in some cases. Indeed, despite the successes of PD-(L)1 inhibitors in various tumors, only a fraction of PD-L1+ cases may benefit from immunotherapy, while some PD-L1- tumors respond to PD-1/PD-L1 inhibitors [7,8,9].

In PCs, insensitiveness to the immune checkpoint blockade may be due to various mechanisms, including modifications of drug targets, activation of pro-survival pathways, disability of apoptosis machinery, relatively low tumor mutation burden and/or scant intratumoral CD8+ T-cell infiltrate. In particular, the paucity of cytotoxic T lymphocytes can be due to several factors, including the presence of suppressive regulatory T-cells and/or myeloid-derived suppressor cells. In other parts of our systematic literature review, we have delineated the intracellular signaling pathways and interactions of the various tumor microenvironment components in affecting the PD-1/PD-L1 axis in PC [10,11,12,13,14,15,16,17,18,19,20,21,22,23,24,25,26,27,28,29,30,31,32,33,34,35,36,37,38,39,40,41,42,43,44,45,46,47,48,49,50,51,52,53,54,55,56,57,58,59,60,61,62,63,64,65,66,67,68,69,70,71,72,73,74,75,76,77,78,79,80,81,82,83,84,85,86,87,88,89,90,91,92,93,94,95,96,97,98,99,100,101,102,103,104,105,106,107,108,109,110,111,112,113,114,115,116,117,118,119,120,121,122,123,124,125,126,127,128,129,130,131,132,133,134,135,136,137,138,139,140,141,142,143,144,145,146,147,148,149,150,151,152,153,154]. 

Experimental studies investigating the effects of various types of treatments (such as checkpoint inhibitors, cancer vaccines, hormonal therapy, radiotherapy, DNA-damaging agents, and chemotherapy) alone or combined, may discover how to overcome the resistance to immunotherapy in PCs: they are important pre-clinical tests to verify the potential inclusion of new therapeutic approaches in clinical practice [59,73,154,155,156,157].

In this part of our systematic review, we’d like to delineate the landscape of pre-clinical studies (including cell lines and mouse models) that tested experimental treatments having effects on the PD-L1 signaling.

## 2. Results

### 2.1. Literature Review Results

**Figure 1:**
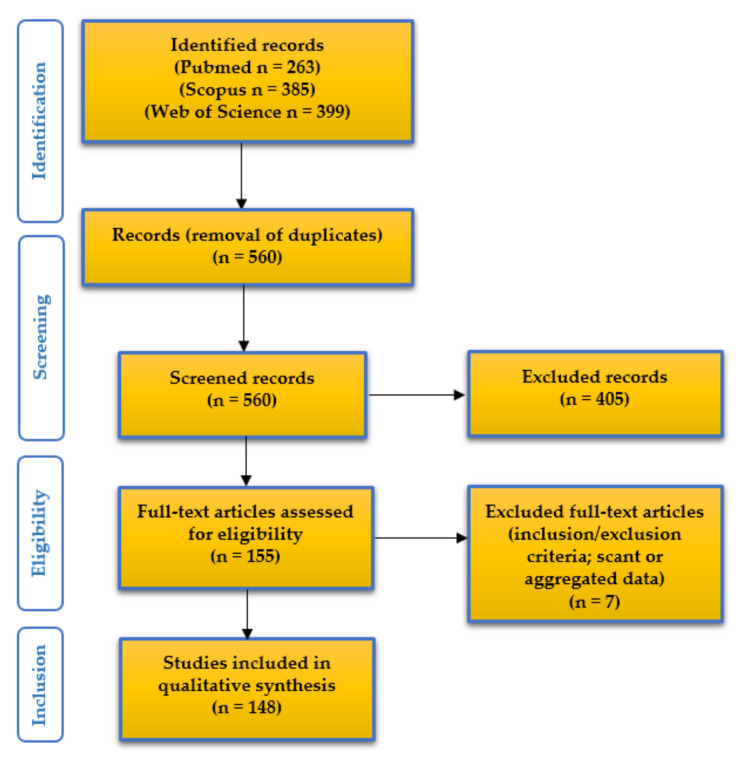
Review of the literature: PRISMA flow-chart.

Figure 1 presents the “Preferred Reporting Items for Systematic Reviews and Meta-Analyses” (PRISMA) (http://www.prisma-statement.org/; accessed on 8 May 2021) flow chart, summarizing the method and results of our systematic literature review.

We identified 263 articles on Pubmed (https://pubmed.ncbi.nlm.nih.gov; accessed on 8 May 2021), 385 articles on Scopus (https://www.scopus.com/home.uri; accessed on 8 May 2021), and 399 articles on Web of Science databases (https://login.webofknowledge.com; accessed on 8 May 2021). After duplicates exclusion, titles and abstracts of the 560 identified records underwent first-step screening: 155 full texts were considered eligible and, after reading them, seven papers were excluded for being unfit according to the inclusion/exclusion criteria, or for presenting scant or aggregated data. One hundred and forty-eight articles were finally included in our study [1,4,7,10,11,12,13,14,15,16,17,18,19,20,21,22,23,24,25,26,27,28,29,30,31,32,33,34,35,36,37,38,39,40,41,42,43,44,45,46,47,48,49,50,51,52,53,54,55,56,57,58,59,60,61,62,63,64,65,66,67,68,69,70,71,72,73,74,75,76,77,78,79,80,81,82,83,84,85,86,87,88,89,90,91,92,93,94,95,96,97,98,99,100,101,102,103,104,105,106,107,108,109,110,111,112,113,114,115,116,117,118,119,120,121,122,123,124,125,126,127,128,129,130,131,132,133,134,135,136,137,138,139,140,141,142,143,144,145,146,147,148,149,150,151,152,153,154]. 

### 2.2. Experimental Studies Tested Various Types of Treatment on PC-Cell Lines to Evaluate Their Effect on PD-L1 Expression/Regulation

As reported in another part of our systematic literature review (see Section 4) [1,4,7,10,11,12,13,14,15,16,17,18,19,20,21,22,23,24,25,26,27,28,29,30,31,32,33,34,35,36,37,38,39,40,41,42,43,44,45,46,47,48,49,50,51,52,53,54,55,56,57,58,59,60,61,62,63,64,65,66,67,68,69,70,71,72,73,74,75,76,77,78,79,80,81,82,83,84,85,86,87,88,89,90,91,92,93,94,95,96,97,98,99,100,101,102,103,104,105,106,107,108,109,110,111,112,113,114,115,116,117,118,119,120,121,122,123,124,125,126,127,128,129,130,131,132,133,134,135,136,137,138,139,140,141,142,143,144,145,146,147,148,149,150,151,152,153,154], the experimental studies performed on human and mouse PC-cell lines revealed that the intracellular ERK/MEK, Akt-mTOR, NF-kB, WNT, and JAK/STAT pathways were involved in PD-L1 expression in PC, usually leading to PD-L1 upregulation. Here, we confirm these results, as the treatment of PC-cell lines with NF-kB, MEK, JAK, or STAT inhibitors down-modulated the expression of PD-L1 (Table 1).

PD-L1 upregulation by tumor cells allows cancers to escape from the antitumor immunity, favoring intratumoral T cells dysfunction by interaction with PD-1 on T cells. To identify extracellular regulators of PD-L1 expression, human and mouse, primary and metastatic cancer cell lines have been treated with blocking antibodies, cytokines, chemokines, radiation therapy and drugs: cell lines were cultured alone or in co-culture with cells of the immune system.

Functional studies revealed that, when PD-L1 is downregulated, inhibited, or blocked in PC-cells, a reduction of both chemoresistance and tumor cell migration occurs. In PC-cells co-cultured with NK, CD8+ T-cells or chimeric antigen receptor T cells (CAR-T cells), the immune cell cytotoxicity activity increased when PD-L1 was downregulated, inhibited, or blocked. Opposite effects were described for PD-L1 upregulation or activation.

A nuclear form of PD-L1 has been recently reported, supporting an additional non-immunogical role of PD-L1. Indeed, nuclear PD-L1 is involved in the regulation of sister chromatid cohesion, genomic stability and pyroptosis [158]. To our knowledge, no functional studies investigating this topic have been conducted on PC cell lines. However, as better described in other parts of our review, Satelli et al. reported that nuclear expression of PD-L1 by circulating tumor cells was associated with worse progression free survival in PC patients [91]. In another clinical study (*n* = 171) [13], nuclear PD-L1 positivity was more frequent in PCs of higher stages, despite it was not predictive of biochemical recurrence free survival. Chemotherapy may induce nuclear translocation of PD-L1, suggesting that this marker has functions other than T cell inhibition [159,160].

### 2.3. Experimental Studies Tested Various Types of Treatment on PC-Mouse Models to Evaluate Their Effect on PD-L1 Expression/Regulation

Data obtained from mouse models revealed that amphiregulin (AREG) was produced by the tumor stroma of PC after damage (i.e., genotoxic chemotherapy and ionizing radiation), conferring resistance to immunosurveillance by increasing PD-L1 expression on cancer cells [120]. Radiotherapy, CDK4/6-inhibitors, and *RB* deletion can induce PD-L1 upregulation, thus causing the immune evasion of PC-cells. Besides, a small bioactive S249/T252 phosphorylation-mimetic peptide of RB can decrease PD-L1 expression via NF-kB inhibition and by enhancing the anti-cancer efficacy of radiotherapy [53].

Regarding inflammation, IL-17 has been proven to promote the infiltration of PD-1+ immune cells into the prostatic stroma, as well as to increase the PD-L1 and PD-L2 expression by tumor cells. Overall, IL-17 wild-type mice developed more invasive PCs than IL-17 knockout mice in the *PTEN*-null background [149]. 

The JAK/STAT pathway is frequently overactivated in PC-cells, and it can trigger PD-L1 upregulation. Indeed, STAT3 plays a key role as a mediator of tumor immune evasion. Adenoviral vectors expressing SOCS3 gene have been shown to increase the sensitivity of PC-cells with JAK/STAT3 overactivation to NK cells, by decreasing PD-L1 expression and IL-6 production [125]. Local administration of CpG-STAT3 antisense oligonucleotides (inhibiting STAT3 and activating TLR9) has been shown to induce systemic antitumor effects in two genetically modified mouse models of PC, by decreasing PD-L1 [129]. It has been also demonstrated that the block of LIF/JAK/STAT signaling with EC330 (LIF inhibitor) in a xenograft mouse model of PC significantly decreased the tumor volume and coupled with a reduction of PD-L1 expression in tumor tissue [104]. Moreover, the N-cadherin antagonist ADH-1 promotes the antitumor response of tumor infiltrating lymphocytes (TILs), interfering with the JAK/STAT pathway [14]: CXCL11 and IRF1 were upregulated after using ADH-1.

Another pathway frequently activated in PC is the Akt-mTOR. *PTEN* upregulation—with consequent inhibition of mTOR and PD-L1—has been documented in mice injected with PC-cells overexpressing chemerin (PTEN activator). The AKT-mTOR inhibition and chemerin-induced PD-L1 downregulation significantly reduced the tumor growth [105]. 

Concerning epigenetic drugs, in accordance with the in vitro data on cancer cell lines, the in vivo treatment of mice carrying PC3 and DU145 xenografts with JQ1 (bromodomain inhibitor) downregulated PD-L1 expression [118].

Furthermore, blocking only the PD-1/PD-L1 pathway has limited anti-tumor efficacy. However, synergistic effects in reducing PC-tumor growth were observed by combining the block of PD-1/PD-L1 axis with other approaches, including: Cabozantinib (tyrosine kinase inhibitor) + BEZ235 (phosphoinositide 3-kinase PI3K/mTOR dual inhibitor) [82]; A485 (p300/CBP inhibitor) [112]; MYC inhibitor 361 [161]; anti-RANKL antibody (alone or associated with anti-CTLA4 antibody) [132]; nitroxoline [124]; DNA vaccines [147]; cell vaccines [114,139]; CAR-T cell therapies [102,143,145]; JAK/STAT3 inhibitors [134]; IL-15 [152]; anchor modified IL-15 and anti-CTLA4 antibody [108]; radiation therapy [121]; and Radium-223 + anti-CTLA4 antibody [12]. Moreover, the combination of classical chemotherapy with Cabazitaxel followed by PD-L1 block has shown more efficacy in reducing tumor growth in comparison to the PD-L1 block followed by Cabazitaxel administration [117]. Finally, it has been demonstrated that the deletion of the gluconeogenesis regulatory enzyme FBP1 (Fructose-1, 6-biphosphatase) induced an increment of the tumor growth, as well as an increase of the anti-PD-L1 treatment resistance [115]. All these data were derived from models of subcutaneous and orthotopic tumor growth in syngeneic immunocompetent mice, spontaneous prostate carcinogenesis in transgenic immunocompetent mice, and human cancer cell xenografts in immunodeficient mice followed by reconstitution with the human immune system (humanized mouse model). In Table 2 and Table 3, the experiments performed in syngeneic and spontaneous PC mouse models are summarized, respectively; unfortunately, few data of humanized mouse models are available [14,120,134]. 

Finally, few articles have investigated the role of PD-1/PD-L1 axis on immune cells in PC mouse models. A study revealed that treatment with oxaliplatin can increase the PD-L1+ tumor-infiltrating B-cells, inducing CD8+ T-cell exhaustion and chemotherapy resistance [95]; another research correlated the resistance to radiotherapy with the increased expression of PD-1 in infiltrating CD45+/CD8+ T-cells [106]. Instead, circulating dendritic cells expressing PD-L1 are involved in resistance to Enzalutamide, an anti-androgen drug used for the treatment of castration-resistant PC (CRPC)-patients [96]. Finally, another study had shown that anti-PD-1 treatment in association with inhibition of the methyltransferase EZH2 induced an increase of intratumoral activated CD8+ T cells and M1 tumor-associated macrophages (TAMs), reducing tumor growth [154].

## 3. Discussion

### 3.1. Inhibitors of PD-L1/PD-1, JAK/STAT, ERK/MEK, Akt-mTOR, NF-kB Pathways, and Cytokines

**Figure 2:**
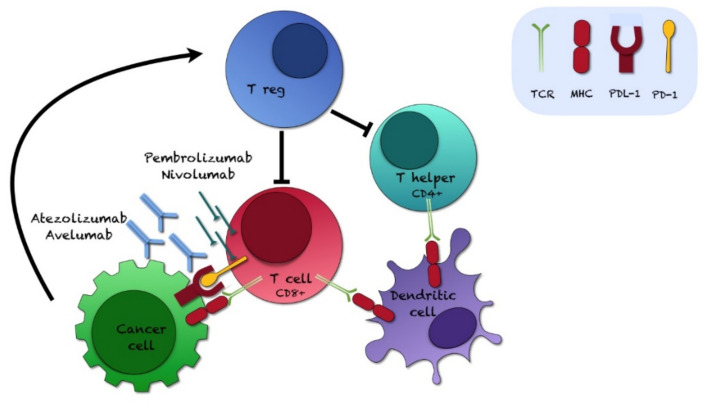
Inhibitors of PD-1 (Pembrolizumab, Nivolumab) and PD-L1 (Atezolizumab, Avelumab). MHC: major histocompatibility complex; TCR: T-cell receptor; T reg: regulatory T cell.

The PD-L1/PD-1 pathway physiologically cooperates in the maintenance of T cell immune homeostasis and peripheral tolerance, preventing T cell hyperactivation and autoimmune responses [158]. To evade the antitumor immunity, cancer cells upregulate PD-L1, which interacts with its receptor (PD-1) on T lymphocytes, causing cytotoxic T cell dysfunction; moreover, other tumor-infiltrating immune or stromal cells may favor immunosuppression [158]. As expected, PD-1 and PD-L1 inhibitors block the effects of their respective targets, reducing the possibility of cancer cells to escape from the antitumor immunity (Figure 2). 

**Figure 3:**
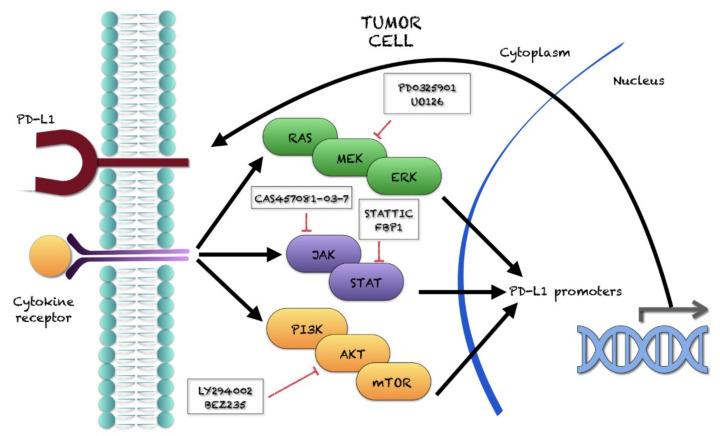
Inhibitors of the RAS/MEK/ERK, JAK/STAT and PI3K/AKT/mTOR signaling pathways are usually negative regulators of PD-L1 expression.

The ERK/MEK, Akt-mTOR, NF-kB, and JAK/STAT signalings upregulate PD-L1 expression, while inhibitors of these pathways have opposite effects (Figure 3). 

Explanations of PD-1/PD-L1 interaction are provided in another part of our systematic review (see Section 4) [1,4,7,10,11,12,13,14,15,16,17,18,19,20,21,22,23,24,25,26,27,28,29,30,31,32,33,34,35,36,37,38,39,40,41,42,43,44,45,46,47,48,49,50,51,52,53,54,55,56,57,58,59,60,61,62,63,64,65,66,67,68,69,70,71,72,73,74,75,76,77,78,79,80,81,82,83,84,85,86,87,88,89,90,91,92,93,94,95,96,97,98,99,100,101,102,103,104,105,106,107,108,109,110,111,112,113,114,115,116,117,118,119,120,121,122,123,124,125,126,127,128,129,130,131,132,133,134,135,136,137,138,139,140,141,142,143,144,145,146,147,148,149,150,151,152,153,154]. Here we focus on some comments about the topic of this paper.

Experimental models have found that combined STAT3 inhibition/TLR9 stimulation in myeloid cells helps in the eradication of solid tumors through efficient recruitment of innate and adaptive effector cells (neutrophils, CD8+/CD4+ T cells) [129]. Broadly specific tyrosine kinase inhibitors (sunitinib, cucurbitacin B, etc.), in vitro STAT3 inhibition, PI3K inhibitors (BEZ235, cabozantinib) may block the JAK/STAT3 signaling and overcome the immunosuppression mediated by myeloid derived suppressor cells (MDSCs) [162]. STAT3 seemed to reduce the antitumor activity of CD8+ T cells and to expand the tumor-promoting Th17 lymphocytes, and it was also important for generating memory T-cells and long-term antitumor immunity [129]. Cell-selective strategies are required, as targeting JAK1/2 kinases upstream from STAT3 reduced MDSCs, but paradoxically increased their immunosuppressive activity, inhibiting the T-cell proliferation [129]. PI3K and PI3K/mTOR inhibitors can interfere with T-cell activation, inducing tolerance [129]. 

Decoy and antisense oligonucleotides inhibiting of the STAT3 signaling (STAT-Oli) were promising in phase I clinical trials [163,164]. Lack of cell-selectivity and targeted delivery of oligonucleotides reduce the efficacy and penetration into the tumor microenvironment. Using human and mouse cellular targets in vitro and in two syngeneic models of bone-localized PCs, some authors found that conjugation of CpG oligodeoxynucleotides (a synthetic TLR9 ligand/agonist) with chemically modified STAT-Oli molecules (CpG-STAT-Oli) may improve targeting of human and mouse PC-cells, and disrupt MDSCs; moreover, it could increase the nuclease resistance, potentially being suitable for systemic administration [129].

Inhibition of the ATM/JAK/PD-L1 signaling pathway may suppress the epithelial–mesenchymal transition (EMT) and metastatic progression of CRPC cells. With the increase of Gleason score, PC-cells gradually loose the structure and basement membranes, forming cell clusters or single cells and rapidly becoming more invasive: the expression of cytokeratins and E-cadherin is downregulated, while mesenchymal cell markers (N-cadherin and vimentin) levels increase [130]. PD-L1 is involved in the EMT of some tumor types. In an experimental study, PD-L1 antibodies and JAK inhibitor 1 significantly decreased the migration of cells and normalized the overexpression of EMT-associated marker genes [130]. Ataxia telangiectasia mutated kinase (*ATM*) gene has a role in cell growth and DNA damage: Zhang et al. found that *ATM* expression was higher in CRPC tissue samples (vs. hormone-dependent PCs) and *ATM* knockout cells induced PD-L1 downregulation [130]. 1/3 responders to Enzalutamide plus Pembrolizumab showed an *ATM R1618Q* mutation [22,90].

Androgen withdrawal may increase tumor inflammation, and mediate the recruitment and accumulation of immunosuppressive cells such as regulatory T cells, M2-polarized macrophages, and MDSCs. Combined treatment with anti-PD-L1 antibody (clone D265A, mouse/IgG1 kappa) plus AZD1480 (JAK1/2 inhibitor) followed by androgen deprivation therapy improved antitumor immune responses over monotherapy in *PTEN*-knockout mice, and it could decrease the immunosuppressive effects of androgen withdrawal. In the study of De Velasco et al. [141], flow cytometry showed post-treatment abrogation of PD-L1 expression in circulating dendritic cells in all settings. Increased numbers of circulating effector memory CD8+ T cells and CD355+/CD8+ T cells were identified, while increased CD8+ T cells and reduced CD25+/CD4+ regulatory T cells were found in tumors. 

Receptor activator of NF-kB ligand (RANKL) and its receptor, RANK, are members of the tumor necrosis factor (TNF) and TNF receptor (TNFR) superfamilies, playing a role in T cell and dendritic cell interactions (with potential immune checkpoint functions), but also in bone homeostasis [132]. Human IgG2 anti-RANKL antibodies (denosumab) have been developed as an anti-resorptive therapy in patients with bone metastases [132]. Ahern et al. found that blockade of RANKL improves the anti-metastatic activity of antibodies targeting PD1/PD-L1, improving tumor growth suppression in PC-mouse models [132].

The “Src homology region 2-containing protein tyrosine phosphatase 2” (SHP2) is a ubiquitous tyrosine phosphatase, activating the signal transduction (including the JAK/STAT pathway) of various growth factors and cytokines: it may act as an oncoprotein (promoting proliferation and survival), but also as a tumor suppressor in some cancers [142,165]. High SHP2/STAT3 (phosphorylated or not) and low SHP1/STAT1 (phosphorylated or not) expression were reported in PC cell lines. 

Major histocompatibility complex (MHC) class I molecules facilitate the immune recognition of cancer cells, promoting the presentation of small-peptide fragments of non-self antigens on the cell surface, allowing their identification by CD8+ cytotoxic T lymphocytes [118,142,166,167,168,169,170]. Like other cancers, PC tumor cells commonly downregulate the MHC class I expression to evade immune detection [118,166,168,169,170].

At least in some PC cell lines, SHP2 is a negative regulator of HLA-ABC and PD-L1 expression via STAT1 phosphorylation, and an activator of the extracellular signal-regulated kinase (ERK) phosphorylation. Pre-treatment with JAK2-inhibitor failed to induce HLA-ABC and PD-L1 expression, while treatment with the mitogen-activated protein kinase/extracellular signal-regulated kinase (MEK) inhibitor PD0325901 did not upregulate HLA-ABC and PD-L1. SHP2 depletion was associated with increased T-cell activation by co-culture of allogeneic healthy donor peripheral blood monocytes with SHP2 siRNA-pretreated tumor cells [142].

Anti-IL-6 antibodies downregulated PD-L1 expression [133,134,135]. IL-17 and TNF-α secreting Th17 cells were enriched in PCs: they may favor an immunosuppressive tumor microenvironment. Through activation of the NF-kB signaling and in the presence of AKT activity, IL-17 and TNF-α may act individually (rather than cooperatively) to upregulate PD-L1 expression in some PC cell lines (LNCaP cells), but only TNF-α induced PD-L1 mRNA levels. NF-kB or AKT inhibitors could diminish the IL-17/TNF-α-induced PD-L1 protein levels. Neither IL-17 nor TNF-α promoted PD-L2 mRNA or protein expression [144,171]. Analyzing PCs of *PTEN*-null mice, Yang et al. found that IL-17rc wild-type mice showed higher levels of PD-1, PD- L1, and PD-L2, developing more invasive PCs than IL-17rc knockout mice [149].

In a PC mouse model, the simultaneous administration of IL-15, anti-CTLA-4, and anti-PDL-1 was associated with increased number of CD8+ T cells, T cell lytic activity, and IFN-γ release, decreased tumor growth, and improved mice survival (compared to IL-15 alone) [152]. PD1 inhibits PI3K activation, while CTLA-4 preserves the PI3K activity but inhibits AKT phosphorylation. This synergistic triple combination therapy directly restored the responsiveness of CD8+ T cells, indirectly inhibiting the suppressive regulatory T cells (Tregs): by targeting different pathways, it led to *AKT* activation [152,172]. 

The cytokine-induced Src homology (SH2)-containing protein (CIS)/Suppressor of cytokine signaling (SOCS) family consists of eight intracellular proteins (CIS and SOCS1-7) [125]: SOCS1 and SOCS3 are involved in cytokine signal control, negatively regulating the activated JAK/STAT signaling in normal cells [125]. Conversely, JAK/STAT over-activation and SOCSs silencing are frequently observed in various cancers [125]. CIS, SOCS1, and SOCS3 proteins may regulate T cells and macrophages activity [125]. Deletion of SOCS3 in T cells and macrophages induced anti-tumor effects in MC38 colon cancer and B16F10 melanoma mouse models [125]. Blocking JAK/STAT3 signaling with SOCS3 might activate antitumor immunity in the tumor microenvironment. Human CRPC androgen receptor (AR)-negative cell lines (DU-145 and PC-3) expressed high levels of IL-6 [173], while the *STAT3* gene was completely deleted (PC-3 cell line) [125]. The replication-deficient recombinant adenoviral vectors Ad-SOCS3 can inhibit cell growth in CRPC cell lines expressing phosphorylated STAT3 (human DU-145 and mouse TRAMP-C2), but not in the human PC-3 CRPC cell line with *STAT3* gene deletion. It could induce the G0/G1 cell cycle arrest by the suppression of STAT3 expression. Ad-SOCS3 could inhibit IL-6 production in DU-145 cells and IFN-γ-induced PD-L1 expression in TRAMP-C2 cells, increasing the NK cell sensitivity of DU-145/TRAMP-C2 cells [125]. Ad-SOCS3 revealed synergistic antitumor effects, if combined with NK cells in a DU-145 xenograft tumor model.

PC can be associated with abnormal cholesterol metabolism and hypercholesterolaemia: the low-density lipoprotein receptor-related protein (LRP) family regulates lipid metabolism by receptor-mediated lipoprotein endocytosis. LRP1 and LRP5 could promote PC-progression. LRP11 upregulation in PC-cell lines activates β-catenin signaling, causing PD-L1 expression independently from the AR status. In addition, LRP11 induced immunosuppression in a co-culture system. LRP11 effects could be inhibited by LRP11 or PD-L1 antibodies, but their therapeutic potential has to be further investigated in vivo [116].

### 3.2. Poly (ADP-Ribose) Polymerase (PARP) Inhibitor

Olaparib (PARP inhibitor) induced the NK-mediated lysis of PC cell lines: this effect was significantly increased by combining Olaparib and Cetuximab (anti-EGFR monoclonal antibody). PARP inhibitors activate the Stimulator of Interferon Genes (STING) pathway, thereby upregulating PD-L1. STING expression was not found in *BRCA* mutant 22RV1 DU145 PC cell lines (either before or after Olaparib treatment), while STING was upregulated after Olaparib exposure in *BRCA* wild-type DU145 cells lines [126]. Olaparib did not induce a significant increase in PD-L1 expression in DU145 cells, but it can enhance the tumor lysis promoted by high-affinity NK cells or NK cells treated with an IL-15/IL-15 receptor-α superagonists [126]. Further data on PARP-inhibitors and “epigenetic” drugs (bromodomain inhibitors, histone-deacetylase/pan-deacetylase inhibitors, etc.) are discussed in other parts of our review (see Section 4).

### 3.3. Indoleamine 2,3-dioxygenase (IDO)

IDO is an enzyme catalyzing the rate-limiting step of Tryptophan (Trp) metabolism to Kynurenine (Kyn) (endogenous ligand for the aryl hydrocarbon receptor), regulating the acquired local and peripheral immune tolerance in physiological and pathological scenarios [49]. IDO is expressed by tumor cells and tumor-associated leukocytes or dendritic cells, inducing T cell dysfunction and apoptosis [150]. In the tumor microenvironment, Trp depletion activates a starvation response in T cells (impairing their function), while Kyn accumulation inhibits the anti-tumor effector T cells, hyperactivating the immunosuppressive Tregs [49]. The production of Kyn and other metabolites favors T-cell G1 arrest, T- and dendritic- cell apoptosis, dampening of NK-cell activity, and enhanced activity of Tregs [58]. IDO expression seems to correlate to shorter survival rates in different cancers, maybe representing a mechanism of immunotherapy-resistance [150]. Carbotti et al. found that IL-27 induced IDO (mRNA and protein) expression at low levels, promoting PD-L1 upregulation in human PC3 PC-cells [150]. 

IDO1 inhibitors may enhance the efficacy of anti-PD-1/PD-L1 drugs, potentiating the action of immune effectors, without directly killing tumor cells or initiating a de novo anti-tumor immune response [49]. A phase Ib study (NCT02471846) [49] enrolled 158 patients to evaluate the effects of the combination of Navoximod (GDC-0919, IDO inhibitor) and Atezolizumab (PD-L1-inhibitor) in locally advanced, recurrent, or metastatic solid malignancies progressing after standard therapy (or for which standard therapy was ineffective, intolerable, or inappropriate). Navoximod + Atezolizumab were active in these patients, showing acceptable safety, tolerability, and pharmacokinetics. Unfortunately, there was no clear evidence of a clinical benefit, and it was unclear how many PC-cases were included.

Zahm et al. assessed IDO activity by serum Kyn/Trp ratios in PC-patients at different stages, being treated with Pembrolizumab (*n* = 8), vaccine (*n* = 10), or both (*n* = 6). IDO activity was associated with a modest decrease in vaccine-induced antigen-specific T-cells, showing the highest levels in patients without benefit from immunotherapy. It increased primarily in patients who did not experience a PSA decline during the 12-week period of treatment. IFN-γ serum concentrations correlated with Kyn/Trp ratios. Biopsies from nine metastatic lesions at baseline and 12 weeks after vaccine ± Pembrolizumab identified IDO staining mainly in myeloid cells/macrophages (CD163+), and not in PC-cells [73]. 

### 3.4. ADAM Inhibitors

The ADAM family includes disintegrin and metalloproteinases with potential adhesion and protease domains. All ADAMs are characterized by a particular domain organization, including a pro-domain, a metalloprotease, a disintegrin, a cysteine-rich, an epidermal-growth factor-like and a transmembrane domain, as well as a C-terminal cytoplasmic tail. They are responsible for the cleavage and/or proteolytic release of various cell-surface proteins, including p75 TNF-receptor, IL-1 receptor type II, TNF-α, E-cadherin, TGF-α, L-selectin, growth hormone receptor, MUC1, and the amyloid precursor protein. High expression of ADAM10 and/or ADAM17 is correlated to unfavorable outcomes and/or treatment resistance in various tumors (biliary, breast, cervical, gastric, hepatocellular, lung, nasopharyngeal, ovarian, pancreatic, skin, urothelial, colorectal, etc.). Other mechanisms of action include enzymatic degradation of the extracellular matrix and tumor cell attachments, alteration of signaling through modification of the surface ligands and receptors (such as Notch, HER2, EGFR, and NKG2D). Metalloprotease inhibitors seem promising in preventing post-radiation resistance in non-small cell lung cancer, and in treating breast cancer. Some authors found that ADAM10 and ADAM17 also cleave PD-L1 to mediate resistance to immunotherapy; however, this role of ADAMs in PC has to be further verified [7].

### 3.5. Fructose-1,6-biphosphatase (FBP1)

In cancers of various organs (breast, liver, kidney, etc.), FBP1 is a putative tumor suppressor, negatively regulating aerobic glycolysis, reducing the Warburg effect and/or antagonizing the function of HIF. FBP1 is often downregulated in many tumor types, and its loss is correlated to tumor progression. FBP1downregulation may be associated with DNA promoter hypermethylation and copy number loss, histone deacetylation (due to histone deacetylase deregulation), or post-transcriptional changes mediated by MAGE-TRIM28, leading to FBP1 degradation in cancer cells [115,174,175].

In PC cell lines, FBP1 inhibited the STAT3-dependent PD-L1 expression: FBP1 competitively sequestered the unphosphorylated STAT3, significantly decreased the STAT3 occupancy on the genomic locus of *CD274* (*PD-L1)* gene, and downregulated the expression of PD-L1. In contrast, ionizing radiation or IL-6 treatment increased the Y705-mediated phosphorylation of STAT3, and impaired the interaction between FBP1 and STAT3, diminishing the inhibitory effects of FBP1 on PD-L1 expression [115,174,175].

### 3.6. Sigma-1 Inhibitors

**Figure 4:**
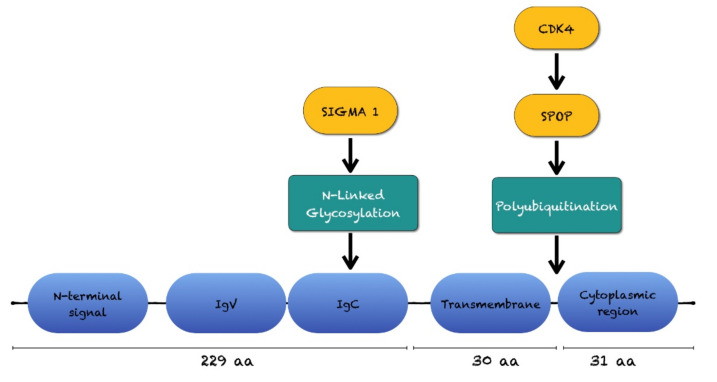
Effects of Sigma-1 and “Speckle Type BTB/POZ Protein” (SPOP) post-translational modifications on the structure of PD-L1 protein (aa: amino acids; CDK4: Cyclin Dependent Kinase 4; IgC and IgV are immunoglobulin-like domains).

The PD-L1 glycoprotein comprises 229 amino acids, including a N-terminal signal sequence, IgV- and IgC- extracellular domains (engaging PD-1 on infiltrating immune cells), a trans-membrane domain, and a relatively short cytoplasmic tail without defined functional motifs (Figure 4) [138,176]. 

The biochemical and molecular mechanisms governing PD-L1 transcription, translation, processing, assembly, transport, and functional binding partners are poorly defined, and few regulatory proteins of PD-L1 have been identified [138,173].

Autophagy represents a set of cellular sequestration and degradation mechanisms by which large aggregates of misfolded proteins and cellular components are sequestered into membrane-bound vesicles (auto-phagosomes), targeted for lysosomal degradation: cells maintain energy levels under metabolic stress through autophagy pathways [138,177,178].

Different autophagy types were described (chaperone-mediated, secretory, or ubiquitin-selective autophagy; bulk macro-autophagy; lipophagy; mitophagy; and endoplasmic reticulum-phagy) [138,177,178]. The secretory membrane remodeling and protein trafficking machinery contributes to the autophagic processes [138,177,178]. 

Sigma1 is a ligand-operated integral membrane chaperone or scaffolding protein highly expressed in the endoplasmic reticulum of various cancer cell lines, being involved in maintaining protein homeostasis and supporting the increased demand for secretory pathways protein synthesis associated with tumor growth [138]. Inhibition of Sigma1 can suppress tumor growth, inducing apoptosis in multiple cancer cell lines [138]. Selective small-molecule modulators of Sigma1 can regulate the protein translation, activating the unfolded protein response and autophagy in pharmacologically controllable settings [138]. In in vitro PC-models, Sigma1 inhibitors—such as 1-(4-Iodophenyl)-3-(2-adamantyl) guanidine (IPAG)—may regulate the transport and stability of PD-L1 in cancer cells, suppressing the IFN-γ-induced PD-L1 surface expression and causing selective autophagic PD-L1 degradation on the endoplasmic reticulum [138].

In an experimental study, Cyclin D-CDK4 kinase destabilizes PD-L1 via culliculin 3-SPOP to control cancer surveillance: treatment of cells with proteasome or ubiquitin E3 ligase inhibitors incremented PD-L1 expression. Cancer-derived SPOP mutants failed to promote PD-L1 degradation by poly-ubiquitination because of their deficiency in binding to PD-L1 [73]. 

### 3.7. Radium-223

Radium-223 is an alpha particle-emitting radiopharmaceutical promoting DNA damages (double-strand DNA breaks) through the release of high linear energy with a range of 100 μm [179]. It is a bone-targeting agent, focusing on tumor-induced osteoblasts by mimicking calcium complexed with hydroxyapatite [180]. Radium-223 may affect tumor cells and tumor microenvironment, improving overall survival (ALSYMPCA phase III clinical trial) [181]. In a pre-clinical study, Radium-223 treatment led to an increase in immune checkpoint modulators including PD-L1 in vitro and in vivo, while plasma-derived exosomes of patients with unfavorable prognosis had higher levels of PD-L1: combining Radium-223 with immunotherapy had greater efficacy than Radium-223 alone [12].

### 3.8. Radiation Therapy

**Figure 5:**
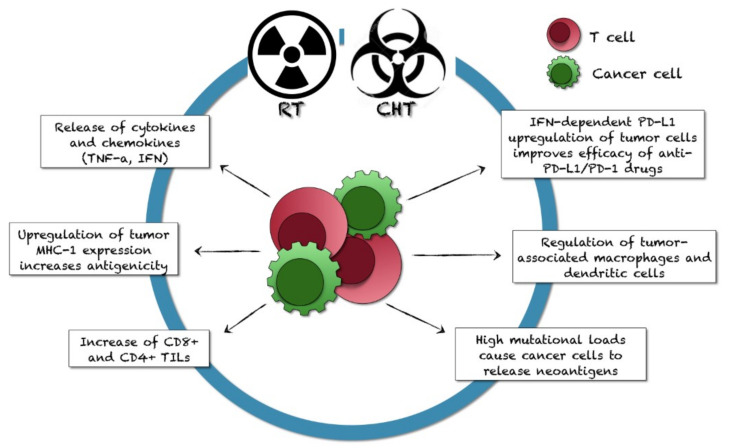
Potential immunogenic effects of Radiotherapy (RT) and/or Chemotherapy (CHT) on tumor cells (IFN: interferon; MHC: major histocompatibility complex; TILs: tumor-infiltrating lymphocytes; TNF: tumor necrosis factor).

Radiotherapy (RT) is indicated in the treatment of selected PC-patients [3]. RT has been previously considered to have an immunosuppressive effect, but new perspectives provided favorable results for combining immunotherapy and RT (Figure 5) [112,182].

RT alters the tumor-cell phenotype, causing DNA damages in tumor cells and mutations in tumor-derived peptides, and increases the release of tumor-associated antigens for the uptake by circulating dendritic cells. So, RT enhances tumor immunogenicity and antigen presentation. Through these mechanisms, RT can activate the adaptive and innate immune systems, causing localized inflammation and increasing the production of inflammatory cytokines, which influence the antitumor immune responses and alter the tumor microenvironment [69]. RT variably modulates different immunosuppressive and immunostimulatory markers, also depending on tumor variability.

The activated immune system may also cause tumor-directed treatment responses away from the site of irradiation (i.e., abscopal treatment effect). In an immune-intact mouse CRPC-model, the median survival was dramatically improved when RT was combined with anti-PD-1 (70% longer) or anti-PD-L1 (130% longer) drugs, respectively (compared to monotherapy) [121].

Fractionated RT induces PD-L1 upregulation through CD8+ T cell production of IFN-γ [69]. Bernstein et al. found that a single RT-dose (10 Gy) enhanced the T-cell cytotoxic activity through increased surface expression of OX40L and 41BBL (tumor necrosis factor superfamily receptors) and decreased PD-L1 expression in three different PC-cell lines (PC3, DU145, LNCaP). However, it failed to reduce the IFN-γ -induced upregulation of PD-L1. CD70 (involved with CD27 in optimal T-cell activation of antigen-naïve T cells) and ICOSL (interacting with ICOS in stimulating proliferation, cytokine production, and effector T-cell generation) increased only in PC3 cells. Normal prostate epithelial cells maintained high PD-L1 expression after irradiation. The immunosuppressor CTLA-4 (expressed on T helper cells) was variably modulated by RT, decreasing (DU145), increasing (LNCaP), or insignificantly increasing (PC3) in three different tumor cells lines [151]. Even a single RT-fraction may increase the total number of tumor-reactive T cells, and the RT-induced overexpression of immunostimulatory molecules (such as OX40L) may favor cytotoxic T CD8+ cells, mitigating the immunosuppressive Tregs.

Antitumor T-cell activation could depend on the relative timing of RT and immunotherapy. In the study of Berstein et al., OX40L and 41BBL were upregulated 72 h post-RT, but these increases were undetectable 144 h following RT. Conversely, the PD-L1 reduction was sustained even after a single RT-dose [151]. 

The radiation-induced changes in immune reaction and expression of costimulatory/coinhibitory molecules in PC-cells seem dose-dependent: low RT-doses favored immune-suppression, while high RT-doses could improve the antitumor ability of the immune system [107,151]. 

Inhibition of the inflammatory response by activating macrophages, enhancement of IFN-γ secretion and antigen-presenting cells (APCs) in the lymph nodes were described after RT administration [107,183]. In some studies, ATR and CHK1 inhibitors attenuated the RT-induced PD-L1 overexpression through the STAT-IRF1-PD-L1 axis [107]. Moreover, the RT-induced high mutational loads may cause cancer cells to release neoantigens, which recruit TILs via a stimulatory signal cascade and promote PD-1/PD-L1 expression in cancer and immune cells [107]. 

Combined treatment with RT plus immunotherapy caused a robust response in pre-clinical studies, with potential PD-L1 inhibition and dendritic cells activation, supporting CD8+ cytotoxic T lymphocytes and mobilizing tumor-specific immunity. However, few preclinical studies are available for PC. In allograft PC-models, 3 x 5 Gy hypofractionated RT can result in tumor growth delay, increased tumor-associated macrophages and dendritic cells, and upregulation of PD-1/PD-L1, as well as of CD8+ T-cell, dendritic cell, and Tregs genes [106,107,121]. In another experimental study, inhibition of S249/T252 phosphorylation by radiation, CDK4/6 inhibitor, or *RB* deletion enhanced PD-L1 expression. A small RB-derived S249/T252 phosphorylation-mimetic peptide overcame the RT-induced immune-tolerance by suppressing PD-L1 expression: it can block the p65 binding to the cognate DNA sequence in the *PD-L1* promoter [53].

The anti-tumor synergistic effect of immunotherapy and RT may be mediated by miRNA regulatory cascades (such as that of the miR-195/-16 family) [69]. miR-195 and miR-16 enhanced the RT-efficacy in PC cell lines, by regulating immunocyte production, activating cytotoxic T cells and reducing regulatory cytokine secretions (such as IFN-γ, TNF-α, and IL-2) in the tumor microenvironment; this synergy was accompanied by the proliferation of functional cytotoxic CD8+ T cells and inhibition of MDSCs and Tregs. Further studies should clarify if RT enhances the innate and adaptive anti-tumor effects of immunotherapy.

### 3.9. Platinum-Based Chemotherapy

PD-1/PD-L1 interaction increases the PC resistance to conventional chemotherapeutic agents (such as doxorubicin and docetaxel) in vitro [148]. Platinum-based drugs are administered for the treatment of various neoplasms (such as pulmonary, ovarian, and colorectal carcinomas) despite considerable toxicity, high incidence of acquired resistance, and limited activity on bone lesions. Moreover, the immunomodulatory profile of these drugs exhibits considerable variability [148].

Oxaliplatin is considered an inducer of immunogenic cell death (ICD) (Figure 5): it activates intracellular stress responses culminating with the emission of adjuvant signals (damage-associated molecular patterns, DAMPs), which initiate the adaptive immunity and regulate cell death in immunocompetent syngeneic settings [113]. Some authors found that mouse B cells modulate the response to low-dose oxaliplatin, promoting tumor-directed CD8+ cytotoxic T cells (CTL) activation: three different mouse PC-models were oxaliplatin-resistant unless genetically or pharmacologically depleted of B cells. The immunosuppressive B cells are represented by plasmocytes expressing IgA, IL-10, and PD-L1 (modulated by TGF-β receptor signaling). Killing these cells allowed the CTL-dependent eradication of oxaliplatin-treated PCs. Oxaliplatin induced Fas ligand (Fas-L) and PD-L1 production in 50% of IgA+ plasmocytes: 40% of them were PD-L1+/IL-10+ [95].

Conversely, the immunogenicity of cisplatin and carboplatin remains a matter of debate. PT-112 is a platinum-pyrophosphate conjugate, specifically created to improve efficacy and limit toxicity. As to its tendency to accumulate in the lung, liver, and bones (in mice), patients with primary or metastatic cancers in these organs, and failing several treatment lines, experienced robust and durable responses upon systemic PT-112 administration in dose-escalation, Phase I clinical trials (NCT02266745, NCT03409458): men with heavily pretreated CRPCs showed serologic and radiographic responses to PT-112, either administrated as monotherapy or combined with avelumab [34,184,185]. In mouse models, PT-112 exerts cytotoxic effects causing the emission of immunostimulatory DAMPs by dying cancer cells: it drives bona fide ICD in vivo, initiating anticancer immunity per se, and synergizing with immunotherapy [113]. PT-112 showed safety profile in heavily pre-treated patients, improving pharmacokinetic and pharmacodynamic features, such as: prominent osteotropism, monotherapy efficacy in pulmonary/prostate cancers and thymoma, combinatorial efficacy in PD-L1 blockage in CRPC, and activity in immunocompetent mouse models of breast and colorectal cancer linked to the initiation of ICD. However, additional studies are required for PC-patients.

Sequential immunotherapy after chemotherapy showed promising potential. However, optimizing synergistic combination and local delivery of effective doses are fundamental. Some authors synthesized ultralarge pore mesoporous silica nanoparticles (UPMSNPs) with anti-PD-L1 antibody (aPD-L1) loaded into the pores: magnetic resonance imaging (MRI)-visible iron oxide ferumoxytol capped the UPMSNP pores. These multifunctional carriers (Fer-ICB-UPMSNPs) were delivered under MRI guidance after a standard cabazitaxel chemotherapy for PC treatment. Cabazitaxel induced ICD, maturation/activation of dendritic cells, tumor-specific T cell proliferation, and PD-L1 upregulation by cancer cell lines. In PC mice models, aPD-L1 loaded on carriers effectively activated T cell infiltration and decreased Tregs. Tumor growth was significantly suppressed, with sequential local delivery of Fer-ICB-UPMSNP after cabazitaxel treatment resulting superior to the systemic immune-checkpoint blockade treatment after the same total dose of Cabazitaxel [117].

### 3.10. Nitroxoline

Nitroxoline showed anticancer activity in breast, bladder, pancreatic, and prostate cancer (as well as myeloma or gliomas) by activating cell apoptosis, arresting cell cycle, and suppressing angiogenesis through MetAP2 activity inhibition. Nitroxoline inhibited the viability and proliferation of mouse PC-cell lines through cell cycle arrest (reduced cyclin D3, CDK2, and CDK6 expression), activation of caspase-3 (major executive apoptotic enzyme), and regulation of apoptosis-related Bcl-2 family proteins [124]. Moreover, nitroxoline inhibited the expression of important proteins of the PI3K/AKT/mTOR pathway, including phospho-PI3 kinase, phospho-Akt (Thr308), phospho-Akt (Ser473), and phospho-GSK-3β. However, no direct evidence supporting the effects of nitroxoline on immune cell function and proliferation was reported. PI3K/AKT/mTOR inhibition may decrease tumor cell proliferation, and enhance tumor immune surveillance by the secretion of immunosuppressive cytokines, the recruitment of intratumoral MDSCs, and the development of memory T cells. AKT controls the balance between terminal differentiation and memory T cell generation, modulating the genesis and differentiation of immunosuppressive MDSCs. In PC mouse models, the combination of nitroxoline and PD-1 blockade increased the number of CD44+/CD62L+/CD8+ memory T cells and reduced the number of MDSCs in peripheral blood, apparently providing synergistic antitumor immunity. Nitroxoline downregulated PD-L1 expression, potentially inhibiting PC-progression [124,186,187,188,189,190,191]. 

### 3.11. Chemokine-like Receptor-1 Inhibitors

Chemerin (or “retinoic acid receptor responder 2”, RARRES2) is an endogenous leukocyte chemoattractant expressed by non-hematopoietic cells, also involved in adipogenesis, metabolism, angiogenesis, and microbial defense. Chemerin is frequently downregulated in various neoplasms, including PC. It recruits inflammatory cells through its G protein-coupled receptor CMKLR1 (Chemokine-like receptor-1), which is expressed by macrophages, dendritic cells, NK cells, and tumor cells [105].

Treatment of PC cell lines with recombinant human chemerin caused a significant increase in *PTEN* expression, while PD-L1 was downregulated [105]. As in PC cell lines, chemerin may act through CMKLR1 on human PC cells to modulate PTEN and PD-L1. Chemerin administration significantly reduced tumor cell migration, increasing T-cell cytotoxicity: the same effects were reported upon *PD-L1* knockdown or treatment with atezolizumab (anti-PD-L1 antibody). *CMKLR1* knockdown or NETA (CMKLR1 small molecule antagonist) administration abrogated the chemerin-induced PTEN and PD-L1 modulation: a potential CMKLR1/PTEN/PD-L1 signaling cascade may occur through the PI3K/AKT/mTOR pathway, as suggested by experiments with targeted inhibitors [105].

### 3.12. Androgen-Deprivation Therapy (ADT)/AR Antagonists

AR is a steroid hormone receptor with a critical role in the signaling pathways of normal prostatic tissue and of PC development/progression, by regulating the transcription of genes involved in cell proliferation, migration, differentiation, cycling, and apoptosis [192]. AR is expressed not only by PC cells, but also by other components of the tissue microenvironment (such as fibroblasts, macrophages, lymphocytes, and neutrophils) [193,194,195,196]. However, as regards cell lines or mouse models, there are limited available data concerning the potential interaction between AR and PD-L1 signalings, as well as regarding the effects of ADT on PD-L1 expression. Further details are also reported in other parts of our review (see Section 4). 

Physical castration or luteinizing hormone-releasing hormone (LHRH) drugs lower the testicular testosterone levels [193]. Moreover, anti-androgens (Bicalutamide, Flutamide and Enzalutamide) directly block the AR function, while CYP17A1 inhibitors (Ketoconazole and Abiraterone acetate) inhibit the extragonadal and intratumoral synthesis of androgens [193]. After early responses, these drugs often become ineffective, and many cases progress to metastatic CRPCs (mCRPCs). PC cells may develop a hypersensitivity to testosterone, activating the AR cascade at castrate levels of circulating hormones. Impaired AR activity in mCRPCs may be due to *AR* gene amplification/mutations, constitutive active AR splice variants, extra testicular testosterone synthesis, overexpression of AR cofactors, and intracrine androgen production [193,194,195,196,197,198,199,200,201,202].

The effects of ADT on the immune system are largely unknown, and those of combined therapy (ADT and immunotherapy; LHRH analogs and AR antagonists) in progressing PCs are still controversial. In a study, Cabazitaxel (AR inhibitor) upregulated PD-L1 in mouse TRAMP-C1 cells [117], while Bicalutamide administration did not change the PD-L1 expression of PC3, DU145 and LNCaP cell lines [94]. Conversely, Bishop et al. described higher PD-L1 levels in Enzalutamide-resistant than Enzalutamide-sensitive LNCaP cell lines [96]. Combined treatment with anti-PD-L1 antibody (clone D265A, mouse/IgG1 kappa) and AZD1480 (JAK1/2 inhibitor) followed by ADT improved antitumor immune responses over monotherapy in *PTEN*-knockout mice, decreasing the immunosuppressive effects of androgen withdrawal [146]. 

As to some studies, ADT/AR antagonists could increase the inflammatory infiltrates, including T cells in peripheral blood of mice and in human PCs [203,204]. However, they may also recruit immunosuppressive cells (such as Tregs, M2-polarized macrophages and MDSCs), potentially impairing the efficacy of immunotherapy [142]. Indeed, some authors reported immunosuppressive effects of ADT on differentiation and activation of T cells, promoting Tregs and TAMs expansion, and counteracting the accumulation of TILs [142,196,205,206,207]. In PC mouse models (Myc-Cap-bearing mice treated with a DC-activating TLR9 agonist), orchiectomy synergized with immunotherapy, while AR antagonists (Flutamide) suppressed the CD8+ T cell reaction (including T cells priming) [205]: the impaired immune response might be correlated with an off-target effect of GABA-A inhibition. These drugs may inhibit the antigen-specific stimulation and the T cell proliferative response to anti-CD3 in a dose-dependent manner (in vitro and in vivo), also decreasing the IL-2 and IFN-γ production by antigen-primed T cells. AR antagonists may activate the immune system (inducing tumor cell apoptosis, thymic enlargement, and leukocytes/B cell migration), and they could exert immunosuppressive effects through an AR-independent pathway [208]. Moreover, neoadjuvant ADT may decrease the number of CD8+ TILs and reduce PD-L1 immunohistochemical expression by tumor cells in PC patients [79].

Proper doses and treatment sequence of AR antagonists and immunotherapy (including vaccines) may improve their synergistic effect on PCs, reducing the immunosuppressive effects of AR antagonists and avoiding the impairment of combined treatment efficacy. Administration of immunotherapy before AR antagonists could have a synergistic impact by temporally inhibiting the suppression of T cell priming. Cabazitaxel treatment followed by PD-L1 blockage more efficiently reduced the tumor growth (if compared to Cabazitaxel admistration after the PD-L1 block) [117]. Further studies are required.

### 3.13. Other Promising Treatment Approaches

CAR-T cell therapy is a novel method of re-engineering native T cells, combining the extracellular antigen recognition domains of a monoclonal antibody and a T-cell receptor activating signaling domain: this technique enhances the antigen-antibody complex formation in response to cytotoxic tumor cell proliferation [189]. So, antigen recognition is not MHC-restricted, like the T cell receptor-mediated antigen identification [145]. Interesting results have been reported in B-cell malignancies [143,209,210], while solid tumors are trickier. 

In castrate metastatic PC, early-phase trials found that CAR-T cell therapy may target the prostate-specific membrane antigen (PSMA), a glycosylated type-II membrane protein that is upregulated in aggressive PCs [145,211]. However, the substantial failure of CAR-T cell response may relate to CAR-T cell inactivation and/or possible exclusion from the tumor mass, tumor-stroma interactions, and PC propensity to metastasize preferentially to the bone. Moreover, CAR-T cells may produce proinflammatory cytokines, increasing PD-L1 expression on tumor cells, and impairing the recruitment/sustained activation of effector T cells: immunomodulation is likely required to increase CAR T-cell efficacy against solid tumors [143]. 

In PC cells co-cultured with NK, CD8+ T-cells, or CAR-T cells, an increase of the immune cell cytotoxicity was observed when PD-L1 was downregulated, inhibited, or blocked. However, an experimental study [145] found that anti-human-PSMA CAR-T cell monotherapy of Myc-CaP (murine PC cell lines) PSMA+ tumors was ineffective, while the combination of anti-human-PSMA CAR-T cells and anti-human-PD1 murine antibody provided a short-duration, sub-optimal response to therapy. 

Intratumoral treatment with armed oncolytic adenoviral vectors expressing immunomodulatory molecules (AOAV) has shown some clinical benefit with a safe profile in localized solid tumors; conversely, the effect against metastasized cancers is limited [143]. Moreover, AOAVs have low transgene capacity, limiting the antitumor immunity enhancement in case of multiple genetic modifications [143]. However, in PC xenograft models, some authors found that the co-administration of an armed oncolytic adenovirus with a helper-dependent adenovirus expressing a PD-L1 blocking mini-antibody may enhance the antitumor effects of CAR T-cells, producing PD-L1 mini-bodies at the tumor site: further data are required [143].

New pharmacological approaches should be studied to enhance the efficacy of immunotherapy. Polypurine reverse Hoogsteen hairpins (PPRHs) are non-modified single-stranded deoxyoligonucleotides formed by two antiparallel polypurine stretches linked by a pentathymidine loop. They can bind to polypyrimidine domains in the double-stranded DNA (dsDNA) of the promoter or intronic regions of target genes, displacing the fourth strand of the dsDNA and producing a triplex structure with transcriptional disruption, causing gene silencing. An experimental study found that PPRHs silencing of both PD-1 and PD-L1 genes induced the clearance of tumor cells by macrophages in co-culture experiments [128].

## 4. Materials and Methods 

Systematic literature reviews (SLRs) and meta-analyses have become increasingly important in health care as: (1) clinicians read SLRs to keep themselves up-to-date; (2) SLRs are often a starting point for developing clinical practice guidelines or further studies/trials; (3) granting agencies could need the results of SLRs to justify a financial support for research projects. So, impacting health care journals frequently ask contributing authors to conduct their SLRs according to the PRISMA guidelines (http://www.prisma-statement.org/; accessed on 8 May 2021), which include an evidence-based minimum set of items for reporting and are useful for a critical evaluation of the submitted manuscripts. We have conducted our SLR according to these guidelines, searching in multiple databases as previously described in the various topics in which PRISMA guidelines are applicable [212,213,214,215,216,217,218,219,220,221,222,223,224,225,226,227,228,229,230,231,232,233,234,235,236,237,238,239,240,241,242,243,244,245,246]. 

Our study aimed to answer the following PICO (Population, Intervention, Comparison, Outcomes) questions:

- Population: patients, tumor cell lines, or mouse models included in studies concerning the role of PD-L1 in PC;
- Intervention: any type of treatment;
- Comparison: no expected comparisons;
- Outcomes: patient’s status at last follow-up (no evidence of disease, alive with disease, dead of disease), response to therapy, biochemical recurrence-free survival, metastasis-free survival, cancer-specific survival, disease-free survival, clinical failure-free survival, overall-survival, progression-free survival; for experiments on PC cell lines and mouse models, any reported effect on cancer and immune cell migration, proliferation, viability, growth, resistance/response to therapy, cytotoxic/anti-tumor activity, PD-L1 expression, and mice/cell lines survival.

Study design: retrospective observational study (experimental studies, case series/reports, clinical trials).

Eligibility/inclusion criteria: experimental studies (tumor cell lines, mouse models) or clinic-pathologic studies on human patients, concerning the role PD-L1 in PCs.

Exclusion criteria: tumors not arising from the prostate; non-carcinomatous histotypes; studies not examining PD-L1; cases with uncertain diagnosis; review articles without new cases.

Information sources and search strategy: we searched for (PD-L1 AND (prostate OR prostatic) AND (adenocarcinoma OR adenocarcinomas OR cancer)) in Pubmed (all fields), Web of Science (Topic/Title), and Scopus (Title/Abstract/Keywords) databases. No limitations or additional filters were set. The bibliographic research ended on 8 May 2021.

Study selection: two independent reviewers selected the studies using a two-step screening method. In the first step, titles and abstracts were screened to verify the eligibility/inclusion criteria, excluding irrelevant articles. In the second step, full texts of all relevant articles were screened by the two reviewers to: (1) verify study eligibility and inclusion criteria; and (2) avoid duplications of the included cases. Two other authors screened the reference lists to search for additional relevant publications. Finally, two authors checked the extracted data. 

Object of the systematic review: (1) to update and summarize the literature concerning the role of PD-L1 in PC cells; (2) to report any information regarding clinic-pathological features, treatment strategies, and patients’ outcomes.

Data collection process/data items: data collection was study-related (authors and year of study publication) and case-related (tumor stage at presentation, Grade Group, type of specimen, treatment, test methods and results of PD-L1 expression, follow-up and outcomes, experiment type). 

Statistical analysis: the collected data were reported as continuous or categorical variables. Categorical variables were analyzed by frequencies and percentages; continuous variables were summarized by ranges, mean and median values. Time-to-recurrence was the time from primary treatment to disease recurrence. The survival status was the time from primary treatment to the last follow-up. 

To better present our SLR results, and discuss the multiple interesting facets of PD-L1 expression by PC, we have split the presentation and discussion of our results into various articles, representing independent, self-sufficient chapters. They highlight various subtopics, including: PD-L1 immunohistochemical expression in PC cases, with discussion of pre-analytical and interpretation variables; clinical-pathological correlations of PD-L1 expression in PC; genetic and epigenetic regulation of PD-L1; PD-L1 intracellular signaling pathways in PC and influence of the tumor microenvironment; investigated correlations of PD-L1 expression with the status of mismatch repair system, *BRCA*, *PTEN,* and other main genes in PC; PD-L1 expression in liquid biopsy samples; information of clinical trials, etc. We address the Readers to these papers for further details [247,248,249,250].

## 5. Conclusions 

In PC, the PD-1/PD-L1 axis regulates various signaling pathways and it is influenced by extracellular factors. 

NF-kB, MEK, JAK, or STAT inhibitors on human and mouse, primary or metastatic, PC-cell lines variably down-modulated PD-L1 expression, which reduced chemoresistance and tumor cell migration. If PC-cells were co-cultured with NK, CD8+ T-cells, or CAR-T cells, the immune cell cytotoxicity increased when PD-L1 was downregulated: opposite effects were found in case of PD-L1 upregulation. 

In mouse models, radiotherapy, CDK4/6-inhibitors, and *RB* deletion induced PD-L1-upregulation, causing PC-immune-evasion. Epigenetic drugs may decrease PD-L1 expression. In some PC experimental models, blocking only the PD-1/PD-L1 pathway had limited efficacy in reducing the tumor growth. Anti-tumor effects could be increased by combining PD-1/PD-L1 blockade with other approaches (inhibitors of tyrosine kinase, PI3K/mTOR or JAK/STAT3 pathways, p300/CBP; anti-RANKL and/or anti-CTLA-4 antibodies; cytokines; nitroxoline; DNA/cell vaccines; radiotherapy/Radium-223).

Different types of mouse models have been used to determine the role of PD-L1, ranging from spontaneous prostate carcinogenesis models to humanized mouse models with a functional human immune system. Most of the preclinical knowledge regarding the role of PD-L1 in PC and the effects of anti-PD-1/PD-L1 immunotherapy derived from experiments on immunocompetent mice, which received the injection of syngeneic mouse PC cells. This approach has the limit that the human and mouse immune systems are similar but not identical. In addition, the onset and progression of PC in mouse models are not alike to the human disease. Humanized mice with a functional human immune system are similar to a human host, and develop tumors that are very close to the human cancers. They represent a highly valuable preclinical model, frequently employed for in vivo research on human cancer immunology and immunotherapy. To our knowledge, only few studies used humanized mice in the PC setting. The application of these preclinical models in PC studies could help to better understand the effects of various treatments (alone or combined) and may favor the discovery of new ways to overcome the immunotherapy-resistance in PC-patients.

## Figures and Tables

**Table 1:** Experimental treatments involved in the regulation of PD-L1 in prostatic carcinoma (pre-clinical studies).

Treatment	Experiment Type	PC Cell Lines	Effects on PD-L1	Studied Effect
**Drugs**				
Ab anti-PD-L1 [63,128]	Treatment	C4-2, CWR22Rv1	Inact	↓ Cell migration (basal condition), ↑ NK cytotoxicity (hypoxia)
Ab anti-PD-L1 [132]	Treatment of co-culture	C4-2 and NK; CWR22Rv1 and NK	Inact	↑ NK cytotoxicity
Ab anti-PD-L1 [148]	Co-culture and Docetaxel treatment	DU145 and Jurkat	Inact	↓ Docetaxel resistance
Oligonucleotides anti-PD-L1 [128]	Co-culture with transfected tumor cells	PC3 and THP1 macrophages	↓	↓ Cell viability ↑ Apoptosis of tumor cells
Ab anti-PD-L1/PD-L1 minibody [143]	Co-culture after treatment	PC3 and CAR-T	Inact	↑ Ability of CAR-T to kill target cells
Avelumab (PD-L1 inh) [126]	Co-culture after treatment	DU145 and NK	Inact	↓ NK cytotoxicity
Atezolizumab (Ab anti-PD-L1) [105]	Treatment of co-culture	DU145 and T	Inact	↑ NK cytotoxicity
Bicalutamide (AR antagonist) [94]	Treatment	PC3, DU145, LNCaP	=	//
Cabazitaxel (AR signaling inh) [117]	Treatment	TRAMP-C1	↑	//
Olaparib (PARP inh) [126]	Treatment	DU145 and NK	=	//
CAS457081-03-7 (JAK inh) [65]	Treatment of co-culture in hypoxic conditon	C4-2 and NK; CWR22Rv1 and NK	↓	↑ NK cytotoxicity
CAS457081-03-7 (JAK inh) [134]	Treatment of co-culture	C4-2 and NK; CWR22Rv1 and NK	↓	↑ NK cytotoxicity
STATTIC (STAT inh) [65]	Treatment of co-culture in hypoxic conditon	C4-2 and NK; CWR22Rv1 and NK	↓	↑ NK cytotoxicity
STATTIC (STAT inh) [134]	Treatment of co-culture	C4-2 and NK; CWR22Rv1 and NK	↓	↑ NK cytotoxicity
Bay11-7082 (NF-kB inh) [144]	Treatment	LNCaP	↓	//
PD0325901 (MEK inh) [142]	Treatment	PC3, DU145	=	//
LY294002 (PI3K/AKT inh) [134]	Treatment	C4-2, CWR22Rv1	=	//
BEZ235 (PI3K/mTOR inh) [105]	Treatment	DU145	↓	//
RAD001 (mTORC1/2 inh) [105]	Treatment	DU145	↓	//
UO126 (MEK inh) [134]	Treatment	C4-2, CWR22Rv1	↓	//
ADAM10 inh [7]	Treatment	DU145	↑ sPD-L1	//
ADAM 17 inh [7]	Treatment	DU145	↑ sPD-L1	//
MG132 (proteasome inh) [73]	Treatment	C4-2	↑	//
MLN4924 (ubiquitin E3 ligase inh) [73]	Treatment	C4-2	↑	//
IPAG (SIGMA-1 inh) [138]	Treatment of co-culture	PC3 and Jurkat	↓	Disruption of checkpoint activity
JQ1 (bromodomain inh) [123]	Treatment	PC3	↓	↓ Proliferation
JQ1 (bromodomain inh) [118]	Treatment	PC3, DU145, Myc-Cap	↓	//
RVX (bromodomain inh) [118]	Treatment	PC3	↓	//
SAHA (HDAC class I and II inh) [112]	Treatment	PC3, DU145	↑	//
LBH589 (pan-deacetylase inh) [112]	Treatment	PC3, DU145	↑	//
A485 (p300/CBP inh) [112]	Treatment	TRAMP-C2 Ras	↓	//
OIRC-9429 (WDR5 inh) [11]	Treatment	PC3, DU145	↓	//
α-NETA (CMKLR1 antagonist) [105]	Treatment	DU145	↓	//
Nitroxoline [124]	Treatment	RM9-Luc-PSA	↓	↓ Cell viability and colony-forming ability
Radium-223 [12]	Treatment	Myc-Cap	↑	//
**Radiation therapy**				
[151]	Treatment	PC3, DU145	↓	//
[121]	Treatment	MyC-CaP	↑	//
[106]	Treatment	TRAMP-C1	↑	//
[151]	Co-culture after treatment	LNCaP and CD8+ T	↓	↑ CD8+ T cytotoxicity

↑: Upregulation/increase; ↓: Downregulation/decrease; =: No alteration; //: no effect was investigated; AR: androgen receptor; CAR-T: Chimeric antigen receptor T cells; HDAC: histone deacetylase; Inact: Inactivation; inh: inhibitor; PARP: poly ADP-ribose polymerase; PC: prostate cancer.

**Table 2:** Syngeneic mouse models of prostate cancer.

Mouse Background	Mouse Cell Lines	Treatment	Effects on PD-L1	Studied Effect
C57BL/6J [53]	PTEN-CaP8 Tsin empty vector or PTEN-CaP8 Tsin-RL S249D/T252D peptide	Gamma-irradiation (12 Gy) + anti-PD-L1	block	Increased anti-cancer efficacy of radiotherapy
Balb/c [125]	TRAMP-C2 expressing SOCS3	No treatment	reduction	Increased sensitivity to infiltrating NK cells
C57BL/6J [129]	RM9 or PPS	CpG-STAT3	reduction	Systemic anti-tumor effects
C57BL/6J [104]	RM9	EC330	reduction	Reduction of tumor growth
CPPSML (PB-Cre+ PtenL/L p53L/L Smad4L/L) [112]	TRAMP-C2 Ras	Anti-PD-L1 + A485	block	Reduction of tumor growth
Tramp [132]	TRAMP-C1	Anti-PD-L1 + anti-CTLA4 + anti-RANKL	block	Reduction of tumor growth compared to single treatments
C57BL/6J [124]	RM9-Luc-PSA	Nitroxoline + anti-PD-1	block and down regulation	Suppression of tumor growth
C57BL/6J [114]	RM1	Anchored-GM-CSF vaccine + anti-PD-1 + anti-Tim3	block	Increase of CD4+ and CD8+ T cells; suppression of tumor growth and tumor regression
C57BL/6J [139]	RM1	Anchored-GM-CSF vaccine + Anti-mPD-1	block	Increase of infiltrating T CD8+ PD-1+ and T CD8+ IFN-γ+ cells
C57BL/6J [152]	TRAMP-C2	IL-15 + anti-CTLA-4 + anti-PD-L1	block	Reduction of tumor growth and prolongation of mice survival
C57BL/6J [108]	TRAMP-C2	cyto-IL-15 + cyto-CTLA4 + cyto-PD-L1	block	Delay in tumor growth and prolongation of mice survival
FVB [121]	Myc-CaP	Anti-PD-L1 + irradiation	block	Reduction of tumor growth and increase of mice survival
C57BL/6J [12]	TRAMP-C2	Anti-PD-1 + anti-CTLA4 + Radium-223	block	Tumor regression
C57BL/6J [117]	TRAMP-C1	Cabazitaxel followed by anti-PD-L1 or anti-PD-L1 followed by Cabazitaxel	block	Cabazitaxel followed by anti-PD-L1 reduced tumor growth, increasing cytotoxic tumor infiltrating cells
C57BL/6J [115]	PTEN-CaP8 wild type or PTEN-CaP8 Fbp1 KO	Anti-PD-L1	block	Increase of tumor growth and resistence to anti-PD-L1 therapy in Fbp1 silenced tumors
FVB and NSG [161]	Myc-CaP	MYCi361 + anti-PD-1 or MYCi975 + anti-PD-1	block	Reduction of tumor growth

**Table 3:** Spontaneous mouse models of prostate cancer.

Mouse Background	Treatment	Effects on PD-L1	Studied Effect
IL17rc wild type *PTEN*^loxp/loxp^ or IL17rc KO *PTEN*^loxp/loxp^ [149]	No treatment	PD-L1 + tumor cells in IL17rc wild type mice	More invasive tumors in IL-17rc wild-type than Il-17rc KO mice in *PTEN*-null background
CPPSML (PB-Cre+ PtenL/L p53L/L Smad4L/L) [82]	Cabozantininb + BEZ235 + anti-PD-1 + anti-CTLA4	PD-L1 block	Decrease of primary tumor growth and metastasies
HHDII-DR1 (HLA-A2.01/HLA-DR1–expressing, murine MHC class I/II KO) treated with 3-methylcholanthrene [147]	DNA vaccines encoding native or modified SSX2 + anti-PD-L1	PD-L1 block	Increased anti-tumor activity of DNA vaccine. Tumor eradication.
TRAMP B cells KO or TRAMP T CD8 cells KO [95]	Oxaliplatin	PD-L1 increase in B cells	Incrase of PD-L1+ tumor-infiltrating B-cells, induction of CD8+ T-cell exhaustion and chemotherapy resistance
